# Protective Effects of Kirenol against Lipopolysaccharide-Induced Acute Lung Injury through the Modulation of the Proinflammatory NFκB Pathway and the AMPK2-/Nrf2-Mediated HO-1/AOE Pathway

**DOI:** 10.3390/antiox10020204

**Published:** 2021-01-31

**Authors:** Frank Cheau-Feng Lin, Shiuan-Shinn Lee, Yi-Ching Li, Yung-Chuan Ho, Wen-Ying Chen, Chun-Jung Chen, Min-Wei Lee, Kun-Lin Yeh, Stella Chin-Shaw Tsai, Yu-Hsiang Kuan

**Affiliations:** 1 School of Medicine, Chung Shan Medical University, Taichung 40201, Taiwan; frnklin@gmail.com; 2 Department of Thoracic Surgery, Chung Shan Medical University Hospital, Taichung 40201, Taiwan; 3 Department of Parenteral Nutrition, Chung Shan Medical University Hospital, Taichung 40201, Taiwan; 4 School of Public Health, Chung Shan Medical University, Taichung 40201, Taiwan; shinn@csmu.edu.tw; 5 Department of Pharmacology, School of Medicine, Chung Shan Medical University, Taichung 40201, Taiwan; annie@csmu.edu.tw; 6 Department of Pharmacy, Chung Shan Medical University Hospital, Taichung 40201, Taiwan; 7 School of Medical Applied Chemistry, Chung Shan Medical University, Taichung 40201, Taiwan; ych065@csmu.edu.tw; 8 Department of Veterinary Medicine, National Chung Hsing University, Taichung 402204, Taiwan; wychen@dragon.nchu.edu.tw (W.-Y.C.); bill05002@gmail.com (K.-L.Y.); 9 Department of Education and Research, Taichung Veterans General Hospital, Taichung 40705, Taiwan; cjchen@vghtc.gov.tw; 10 A Graduate Institute of Microbiology and Public Health, National Chung Hsing University, Taichung 402204, Taiwan; cat852654@gmail.com; 11 Da Vinci Surgical Center, Tungs’ Taichung MetroHarbor Hospital, Taichung 435403, Taiwan; t5722@ms.sltung.com.tw

**Keywords:** lipopolysaccharide, acute lung injury, kirenol, NF-κB pathway, AMPK2/Nrf2-mediated HO-1, AOE pathway

## Abstract

Acute lung injury (ALI) is an acute and life-threatening inflammatory disease of the lung parenchyma that is associated with high mortality worldwide. No therapeutic strategies have been developed for the mitigation of the proinflammatory response that characterizes ALI. Kirenol has anti-inflammatory, antiarthritic, and immunoregulatory effects. In the present study, we investigated the protective effects of kirenol against lipopolysaccharides (LPS)-induced ALI in mice. Kirenol reduced the LPS-induced histopathology changes involving edema and thickening of the interstitial or alveolar walls, infiltration of leukocytes, formation of hyaline membrane. Pretreatment with kirenol reduced leukocytes infiltration in bronchoalveolar lavage fluid (BALF), the alveolar-capillary barrier disruption and lipid peroxidation in lung tissues induced by LPS. Kirenol significantly inhibited the secretion of cytokines, IL-1β, IL6, and TNFα, into the BALF of the mice with LPS-induced ALI through NFκB activation. Moreover, kirenol attenuated the downregulation of the antioxidant enzymes, superoxide dismutase, glutathione peroxidase, and catalase that was induced by LPS. HO-1 expression and the phosphorylation of Nrf2 and AMPK2 were also induced by kirenol. The results indicate that kirenol can be developed as a treatment strategy for ALI, and its effects are induced through the inhibition of the NF-κB proinflammatory pathway and promotion of AMPK2/Nrf2-mediated HO-1 and antioxidant enzymes (AOE) activation.

## 1. Introduction

Acute lung injury (ALI) is an acute and life-threatening inflammatory disease of the lung parenchyma that is caused by several direct and indirect risk factors. ALI considerably varies in terms of severity, from short-term dyspnea to respiratory failure and acute respiratory distress syndrome [1]. The severe hypoxemia patients with ALI experience are due to histopathological changes, called “alveolar damage” including intra-alveolar hemorrhage, alveolar membrane thickening, leukocyte infiltration and accumulation, hyaline membrane formation which consisting of fibrin, plasma proteins and surfactant [2]. Oxidative stress has been observed in inflammatory lung tissue and contributes crucially to the pathogenesis of ALI. Oxidative stress induced by the overproduction of reactive oxygen species (ROS) and the depletion of antioxidative enzymes (AOE). ROS include nonradical molecules like hydrogen peroxide and singlet oxygen as well as free radicals such as superoxide anion and hydroxyl radical. The content of intracellular ROS expression is regulated by at least two types of AOEs. The first type comprises enzymes are associated with ROS scavenger, containing superoxide dismutase (SOD), glutathione peroxidase (GPx), and catalase [3,4]. The second type comprises oxidative stress-induced enzyme, such as heme oxygenase-1 (HO-1) [4,5]. Expression of SOD, GPx, catalase, and HO-1 is regulated by nuclear factor erythroid 2–related factor 2 (Nrf2), which is the transcription factor and downstream factor of 5’AMP-activated protein kinase-2 (AMPK2). In addition, the intracellular oxidative stress leads to the secretion of proinflammatory cytokines such as interleukin (IL)-1β, IL-6, and tumor necrosis factor alpha (TNF-α) through the activation of nuclear factor (NF)-κB, a proinflammatory transcription factor [6,7,8]. The inflammatory response in ALI is exacerbated by the generation of ROS and proinflammatory cytokines [4,7,8].

To date, no effective therapeutic strategy to reduce the proinflammatory response in ALI, which has high global mortality rates, has been developed [9]. Kirenol is a diterpenoid compound derived from *Herba Siegebeckiae*, which is commonly used in traditional Chinese medicine to treat arthritis, malaria, hypertension, snake bite, fatigue, and headache [10,11,12,13]. Specifically, kirenol constitutes the major active component of *Herba Siegesbeckiae* extract. It has anti-inflammatory, antiarthritic, and immunoregulatory effects [14,15,16]. No study has indicated that kirenol has protective effects on ALI in lipopolysaccharide (LPS)-treated mice. Therefore, the present study evaluated whether kirenol pretreatment protects mice with LPS-induced ALI from histopathological damage and explored the underlying molecular mechanisms.

## 2. Materials and Methods

### 2.1. Materials

Kirenol was purchased from Chemface Biochemical Co. (Wuhan, Hubei, China). Thiobarbituric acid reactive substances (TBARS) assay kit, SOD assay kit, GPx assay kit, catalase assay kit, IL-1β enzyme-linked immunosorbent assay (ELISA) kit, IL-6 ELISA kit, TNF-α ELISA kit were acquired from Cayman Chemical Co. (Ann Arbor, MI, USA). The phosphor (P)-NF-E2-related factor 2 (Nrf2) antibodies was acquired from Abcam Biotechnology Inc. (Cambridge, MA, USA). The P-Adenosine 5’-monophosphate-activated protein kinase 2 (AMPK2) was acquired from Cell Signaling Technology Inc. (Beverly, MA, USA). The AMPK2, Heme oxygenase 1 (HO-1), β-actin, Nrf2, AMPK2 P-p65, p65, and IκB antibodies were obtained from Santa Cruz Biotechnology Inc. (Santa Cruz, CA, USA). Bio-Rad protein assay kit was obtained from Bio-Rad Laboratories (Hercules, CA, USA). Zoletil 50^®^ was obtained from Vibac Laboratories (Carros, France). T-PER^®^ Tissue Protein Extraction Reagent, enhanced chemiluminescence reagents, and bicinchoninic acid (BCA) Protein Assay Kit were Thermo Fisher Scientific (Waltham, MA, USA). Lipopolysaccharide (LPS) isolated from *Escherichia coli* 0111:B4, phosphate-buffered saline (PBS), dimethyl sulfoxide (DMSO) were obtained from Sigma-Aldrich (St. Louis, MO, USA).

### 2.2. Animal Model of ALI

BALB/c male mice weighing 25–35 g were obtained from the National Laboratory Animal Center (Taipei, Taiwan) and were housed in the laboratory. They were put on alternating 12-h cycles of darkness and light under specific pathogen-free conditions and were given free access to food and water. Room temperature was maintained at 22 ± 2 °C. All procedures involving the use of the mice were approved by the Institutional Animal Ethics Committee of Chung Shan Medical University (No. 2408). During the study on the preventive effect of kirenol on ALI induced by LPS, the pathogen-free animals were divided into six groups. The control group, named Group I, received vehicle intraperitoneal (IP) injection for 30 min followed by 24 h intranasal administration of 20 μL of saline using a pipette. Group II received vehicle IP injection followed by the intranasal administration of 100 μg/20 μL LPS. Groups III, IV, and V received IP injection of 30, 50, and 100 mg/kg of kirenol followed by the intranasal administration of LPS, respectively. Group VI received IP injection of 1 mg/kg dexamethasone followed by the intranasal administration of LPS. Study on the therapeutic effect of kirenol on ALI induced by LPS, the pathogen-free animals were divided into three groups. The control group, named Group A, received intranasal administration of saline using a pipette for 6 h followed by 18 h IP injection of vehicle. Group B received intranasal administration of LPS for 6 h followed by 18 h IP injection of vehicle. Group C received intranasal administration of LPS for 6 h followed by 18 h IP injection of kirenol at 100 mg/kg. The mice were euthanized through IP injection of 50 mg/kg Zoletil 50^®^ which containing the mixture of zolazepam and tiletamine hypochloride [4,7,8].

### 2.3. Histopathological Study

At the end of the study, the mice were sacrificed, and their lungs were harvested for histological evaluation. The harvested lungs were collected and fixed with fresh 4% paraformaldehyde buffered with PBS before being dehydrated with graded alcohol, embedded in paraffin at 60 °C, and cut sagittally into 5 μm sections. The sections were stained with hematoxylin and eosin. The histopathological characteristics, including edema and interstitial or alveolar thickening, leukocyte infiltration, and hyaline membrane formation, were examined using light microscopy [4]. The histological scores of each group were recorded from 0 to 4. Score “0” means normal condition and “4” means the severity of the disease. The larger the number was the higher the severity of the disease [17].

### 2.4. Bronchoalveolar Lavage Fluid Collection

After the mice were sacrificed, the tracheostomy was performed and the plastic tube slide was placed into the trachea. A sterile syringe was used to instill 1 mL of precooled and pyrogen-free PBS slowly into the lungs via the tracheal cannula. After the lungs were gently massaged for a few moments, the bronchoalveolar lavage fluid (BALF) was collected using a syringe. This collection process was repeated three times. The BALF was centrifuged at 1000× *g* for 10 min at 4 °C. Next, the content of protein in the BALF supernatant was measured using the Bio-Rad Protein Assay Kit. A cell counter was used in the assessment of leukocyte recruitment into the alveolar space [4,7,8].

### 2.5. Thiobarbituric Acid Reactive Substances Assay

To determine the injury level of oxidative stress, we performed lipid peroxidation detection. The degree of lipid peroxidation was assessed based on the presence of thiobarbituric acid (TBA) reactive substances (TBARS) in the lung homogenates [4]. After lung sonication and homogenization, the lysates were incubated with 10% ice-cold trichloroacetic acid. After centrifugation 5000× *g* for 10 min, the TBA was added to supernatant. The mixtures were then kept in a boiling water-bath for 10 min and after cooling under tap water. The optical density of the supernatant was measured at 530 nm using the Synergy HT Multi-Mode Microplate Reader (BioTek, Winooski, VT, USA).

### 2.6. Antioxidant Enzyme Capability and Cytokine Generation Assay

The activity of antioxidant enzymes (AOEs), including SOD, GPx, and catalase, in the lung tissue were assessed using commercially available assay kits. The concentrations of TNF-α, IL-1β, and IL-6 in the BALF were determined using commercially available enzyme-linked immunosorbent assay (ELISA) kits. All experimental procedures were carried out according to the manufacturer’s protocols.

### 2.7. Western Blot Assay

After treatment, the total protein in the lungs was extracted using ice-cold T-PER Tissue Protein Extraction Reagent, to which 1 mM dithiothreitol, 1 mM phenylmethylsulfonyl fluoride, and phosphatase inhibitors (containing 50 mM sodium fluoride, 1 mM sodium orthovanadate, 10 mM sodium pyrophosphate, 1 nM microcystin) had been added using an electric homogenizer. After centrifugation at 1000× *g*, the concentration of protein from the lungs was measured by using the bicinchoninic acid (BCA) protein assay kit. The equal amounts of protein in each sample were harvested and incubated in Laemmli sample buffer, which contain 2% sodium dodecyl sulfate, 10% 1,2,3-propanetriol, 5% β-mercaptoethanol, 0.002% bromophenol blue, 67.5 mM Tris-HCl (pH 6.8). After boiling, the samples were separated using 7.5–12.5% sodium dodecyl sulfate-polyacrylamide gel electrophoresis, followed by transfer to the polyvinylidene difluoride (PVDF) membranes. The PVDF membranes were blocked with 5% nonfat milk in PBS containing 0.1% Tween-20 (PBST) for 1 h at room temperature. And then, the membranes were incubated overnight at 4 °C with specific primary antibodies, including P-AMPK2, AMPK2, P-Nrf2, Nrf2, HO-1, P-p65, p65, IκB, and β-actin antibodies. After washing with PBST, the membranes were probed with horseradish peroxidase–conjugated secondary antibody for 1 h at room temperature. Finally, the immunoreactive bands on the membranes were visualized using enhanced chemiluminescence reagents and imaged using the Infiniti Vision System (Vilber, Lourmat, Collegien, France) [4,7,8].

### 2.8. Statistical Analysis

The results from the present study were statistically analyzed using one-way analysis of variance, with Bonferroni posttest correction used for multigroup comparisons. A *p* value of <0.05 was considered significant. The results are presented as means ± standard deviations (S.D.) and were analyzed using SPSS Statistics for Windows, version 14.0 (SPSS Inc., Chicago, IL, USA).

## 3. Results

### 3.1. Kirenol Protected the Lung Histopathological Changes in Mice with LPS-Induced ALI

**Figure 1:**
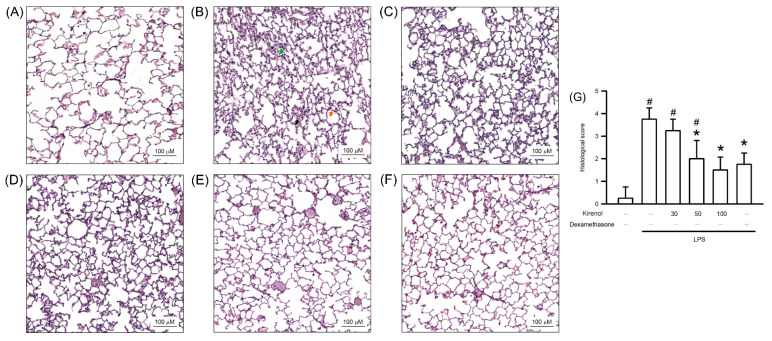
Kirenol protected against histopathological changes in lung tissues in lipopolysaccharide (LPS)-induced Acute lung injury (ALI) mice. (**A**) Control group also named Group I; (**B**) LPS group also named Group II; (**C**) 30 mg/kg kirenol + LPS group also named Group III; (**D**) 50 mg/kg kirenol + LPS group also named Group IV; (**E**) 100 mg/kg kirenol + LPS group also named Group V; (**F**) 1 mg/kg dexamethasone + LPS group also named Group VI. Histopathological changes were observed using Hematoxylin-eosin staining of lung sections under light microscopy. The magnification of the enlargements are 100 times. The scale bars represent 100 μm. Green arrow indicates neutrophil infiltration; black arrow indicates hyaline membrane formation; orange arrow indicates alveolar wall thickness. (**G**) The histologic scores are presented for the lung tissues. Values are shown the mean ± S.D. of 3–4 mice per group. ^#^ represents the significant difference compared with the Group I (*p* < 0.05); * represents the significant difference compared with the Group II (*p* < 0.05).

The effects of kirenol on alleviating ALI induced by LPS were observed in the mice pretreated with kirenol or its solvent for 30 min; these mice then received 24 h intranasal instillation LPS or its solvent. The hematoxylin–eosin staining results, obtained through light microscopy, revealed normal pulmonary histology in the control group. By contrast, the lung tissue in the LPS-treated group showed clear injury, as indicated by edema and interstitial or alveolar thickening, leukocyte infiltration, and hyaline membrane formation. The histopathological changes were inhibited by kirenol in a concentration-dependent manner. In addition, dexamethasone, administered in Group VI, reduced LPS-induced histopathological changes (Figure 1).

### 3.2. Kirenol Protected the Alveolar-Capillary Barrier Disruption in Mice with LPS-Induced ALI

**Figure 2:**
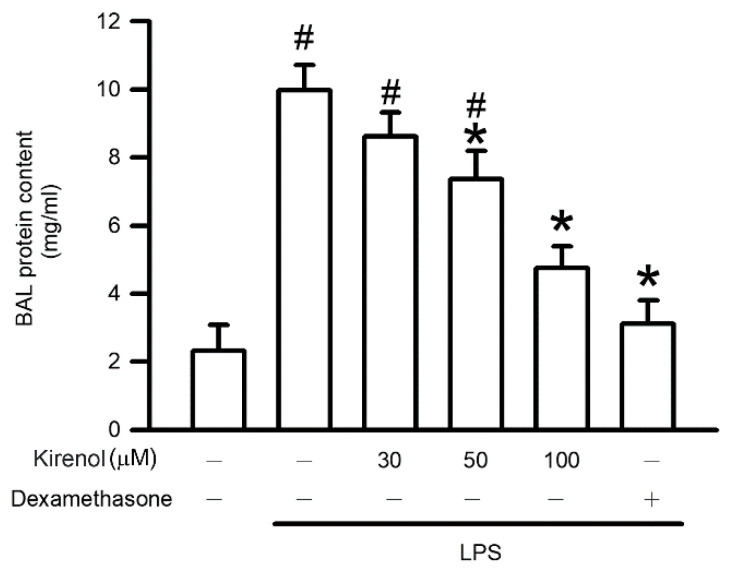
Kirenol protected against LPS-induced alveolar-capillary barrier disruption. The contents of protein in bronchoalveolar lavage fluid (BALF) were measured by Bradford protein analysis. Values are shown the mean ± S.D. of 3–4 mice per group. ^#^ represents the significant difference compared with the Group I (*p* < 0.05); * represents the significant difference compared with the Group II (*p* < 0.05).

Leakage of plasma protein into the alveolar space may indicate LPS-induced alveolar–capillary membrane disruption. The levels of protein content were 2.32, 9.98, 8.61, 7.36, 4.76, and 3.11 at the treatment condition of Group I to VI, respectively. The protein content in the BALF of the LPS-treated group was significantly higher than that in the control group (*p* < 0.05). However, kirenol pretreatment inhibited plasma protein leakage into the alveolar space in a concentration-dependent manner, with significant effects observed starting at 50 mg/kg (*p* < 0.05). Moreover, we also found the mice treatment with dexamethasone attenuated plasma protein leakage after LPS administration (*p* < 0.05; Figure 2).

### 3.3. Kirenol Protected the Leukocyte Infiltration and Lipid Peroxidation in Mice with LPS-Induced ALI

**Figure 3:**
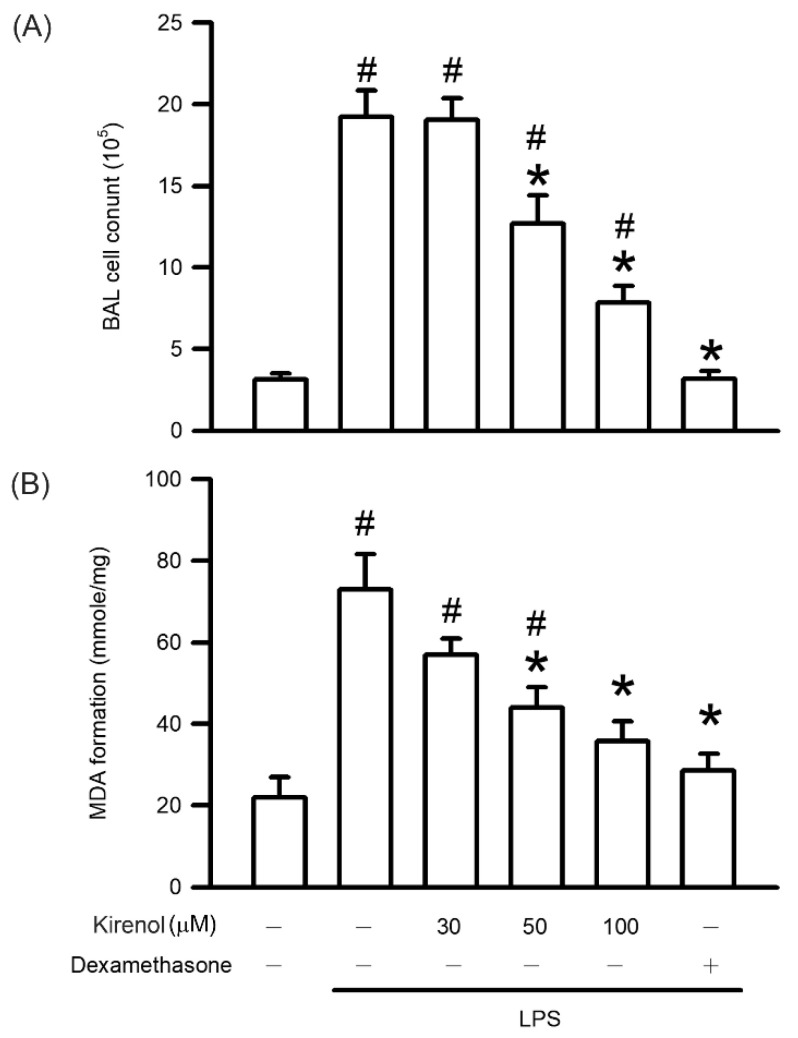
Kirenol protected against leukocyte infiltration and lipid peroxidation. (**A**) Leukocytes infiltration was determined by cell counter assay activity in BALF. (**B**) Lipid peroxidation was determined by thiobarbituric acid (TBA) reactive substance assay in the lungs. Values are shown the mean ± S.D. of 3–4 mice per group. ^#^ represents the significant difference compared with the Group I (*p* < 0.05); * represents the significant difference compared with the Group II (*p* < 0.05).

After activation, leukocytes migrate into the alveolar space, generating considerable oxidative stress, through which lipid peroxidation is initiated. This leads to the disruption of the alveolar–capillary membrane, which in turn facilitates more leukocyte infiltration into the lungs. As shown in Figure 3, the leukocyte numbers in BALF were 3.14, 19.26, 19.06, 12.71, 7.83, and 3.19 × 10^5^ at the treatment condition of Group I to VI, respectively. The content of malondialdehyde (MDA) formation, the product of lipid peroxidation in lung tissue were 22.12, 73.03, 57.09, 44.05, 35.83, and 28.58 mmol/mg at the treatment condition of Group I to VI, respectively. Leukocyte infiltration and lipid peroxidation were significantly higher in the LPS-treated group than in the control group (*p* < 0.05). Kirenol administration reduced the occurrence of both in the mice with LPS-induced ALI in a concentration-dependent manner, with significant effects observed starting at 50 mg/kg (*p* < 0.05). More, we also found the mice treatment with dexamethasone attenuated leukocyte infiltration and lipid peroxidation after LPS administration (*p* < 0.05; Figure 3).

### 3.4. Kirenol Protected the NF-κB p65 Phosphorylation and of IκB Degradation in Mice with LPS-Induced ALI

**Figure 4:**
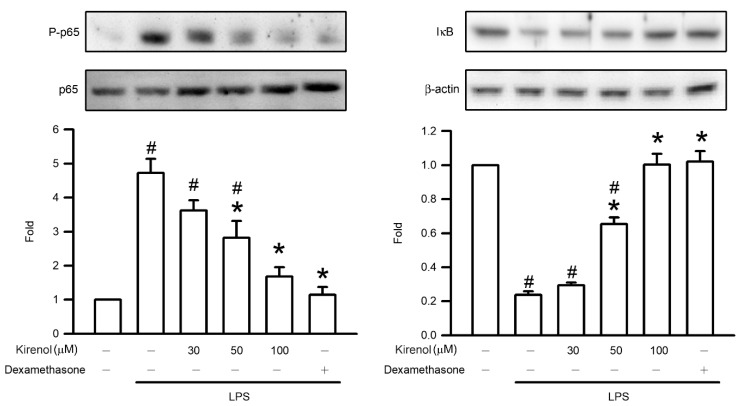
Kirenol protected against the phosphorylation of NF-κB p65 and degradation of IκB induced by LPS. The levels of NF-κB p65 phosphorylation and IκB degradation in the lung tissues were analyzed by Western blotting analysis. Values are shown the mean ± S.D. of 3–4 mice per group. ^#^ represents the significant difference compared with the Group I (*p* < 0.05); * represents the significant difference compared with the Group II (*p* < 0.05).

Activation of the NF-κB pathway induced by LPS or oxidative stress contributes to proinflammatory responses in mice with ALI. In the present study, Western blotting was used to assess the mechanisms of this activation, namely NF-κB p65 phosphorylation and IκB degradation. The fold of NF-κB p65 phosphorylation in lung tissue were 4.72, 3.62, 2.82, 1.68, and 1.14 compared to Group I at the treatment condition of Group II to VI, respectively. The fold of IκB degradation in lung tissue were 0.24, 0.29, 0.65, 1.00, and 1.02 compared to Group I at the treatment condition of Group II to VI, respectively. Phosphorylation of p65 and degradation of IκB in the LPS-treated group was significantly higher than that in the control group (*p* < 0.05). Administration of kirenol inhibited phosphorylation of p65 and degradation of IκB in the mice with LPS-induced ALI in a concentration-dependent manner, with significant effects observed starting at 50 mg/kg (*p* < 0.05) Moreover, we also found the mice treatment with dexamethasone attenuated phosphorylation of p65 and degradation of IκB after LPS administration (*p* < 0.05; Figure 4).

### 3.5. Kirenol Protected the Production of Proinflammatory Cytokines in Mice with LPS-Induced ALI

**Figure 5:**
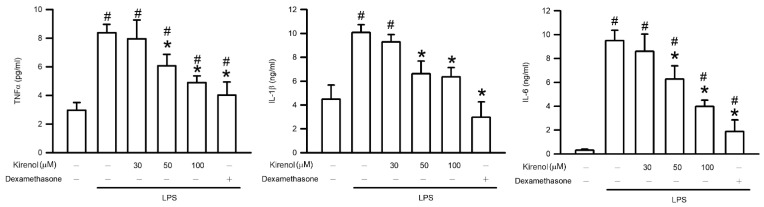
Kirenol protected against LPS-induced generation of proinflammatory cytokines in BALF. Proinflammatory cytokines, including TNFα, IL-1β, and IL-6, were determined by the ELISA assay. Values are shown the mean ± S.D. of 3–4 mice per group. ^#^ represents the significant difference compared with the Group I (*p* < 0.05); * represents the significant difference compared with the Group II (*p* < 0.05).

In mice with ALI, the generation of proinflammatory cytokines such as TNFα, IL-1β, and IL-6 is induced by NF-κB activation. In the present study, the concentration of proinflammatory cytokines were measured through ELISA in the BALF. The concentration of TNFα in BALF were 2.97, 8.37, 7.95, 6.07, 4.90, and 4.03 at the treatment condition of Group I to VI, respectively. The concentration of IL-1β in the BALF were 4.49, 10.08, 9.29, 6.62, 6.36, and 2.97 at the treatment condition of Group I to VI, respectively. The concentration of IL-6 in the BALF were 0.31, 9.50, 8.60, 6.28, 3.97, and 1.88 at the treatment condition of Group I to VI, respectively. The level of proinflammatory cytokines was significantly higher in the LPS-treated group than in the control group (*p* < 0.05). Kirenol administration inhibited the production of proinflammatory cytokines in the mice with LPS-induced ALI in a concentration-dependent manner, with significant effects observed starting at 50 mg/kg (*p* < 0.05). Moreover, we also found the mice treatment with dexamethasone attenuated generation of proinflammatory cytokines after LPS administration (*p* < 0.05; Figure 5).

### 3.6. Kirenol Attenuated the Downregulation of AOEs Activities in Mice with LPS-Induced ALI

**Figure 6:**
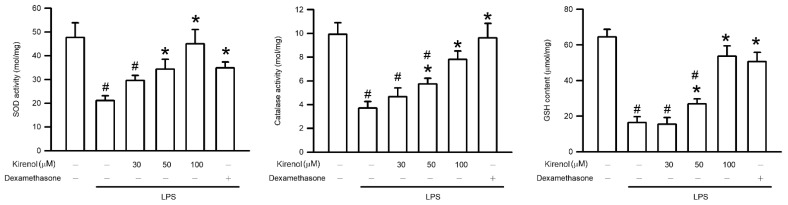
Kirenol attenuated the LPS-reduced the activities of superoxide dismutase (SOD), catalase, GPx. Values are shown the mean ± S.D. of 3–4 mice per group. ^#^ represents the significant difference compared with the Group I (*p* < 0.05); * represents the significant difference compared with the Group II (*p* < 0.05).

Oxygen stress induces tissue damage as a result of the failure of AOEs to detoxify the excessive accumulation of ROS through their endogenous activity [18,19]. Thus, the present study further investigated the effect of kirenol on the activity of AOEs, including SOD, GPx, and catalase. As shown in Figure 6, the level of SOD activity in lung tissue were 47.67, 21.13, 29.61, 34.37, 45.07, and 34.86 mol/mg at the treatment condition of Group I to VI, respectively. The levels of catalase activity in lung tissue were 9.92, 3.72, 4.67, 5.74, 7.81, and 9.62 mol/mg at the treatment condition of Group I to VI, respectively. The level of catalase activity in lung tissue were 64.47, 16.39, 15.41, 26.88, 53.71, and 50.64 μmol/mg at the treatment condition of Group I to VI, respectively. LPS significantly reduced AOE activity in the mice with LPS-induced ALI. Kirenol administration attenuated the decline in AOE activity in a concentration-dependent manner, with significant effects observed starting at 50 mg/kg (*p* < 0.05). Moreover, we also found the mice treatment with dexamethasone attenuated the decline in AOE activity after LPS administration (*p* < 0.05; Figure 6).

### 3.7. Kirenol Enhanced the Nrf2 Phosphorylation and HO-1 Expression in Mice with LPS-Induced ALI

**Figure 7:**
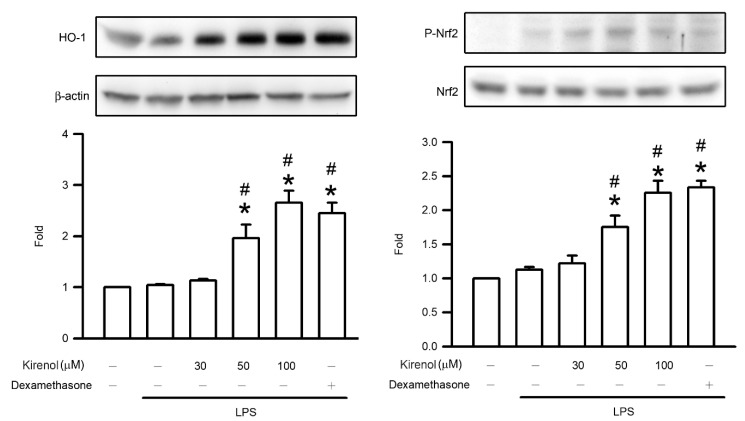
Kirenol enhanced the phosphorylation of Nrf2 and expression of HO-1 induced by LPS. The levels of Nrf2 phosphorylation and HO-1 expression in the lung tissues were analyzed by Western blotting analysis. Values are shown the mean ± S.D. of 3–4 mice per group. ^#^ represents the significant difference compared with the Group I (*p* < 0.05); * represents the significant difference compared with the Group II (*p* < 0.05).

The Nrf2/HO-1 pathway, the upstream signaling pathway of AOEs, involves Nrf2 phosphorylation and HO-1 expression. The fold of HO-1 expression in lung tissue were 1.00, 1.04, 1.13, 1.96, 2.65, and 2.45 compared to Group I at the treatment condition of Group I to VI, respectively. The fold of Nrf2 phosphorylation in lung tissue were 1.00, 1.13, 1.22, 1.76, 2.25, and 2.34 compared to Group I at the treatment condition of Group I to VI, respectively. Nrf2 phosphorylation and HO-1 expression were comparable between the groups treated with LPS and the control group. Administration of kirenol induced Nrf2 phosphorylation and HO-1 expression in a concentration-dependent manner, with significant effects observed starting at 50 mg/kg (*p* < 0.05). Moreover, we also found the mice treated with dexamethasone induced Nrf2 phosphorylation and HO-1 expression after LPS administration (*p* < 0.05; Figure 7).

### 3.8. Kirenol Attenuated the Downregulation of AMPK2 Phosphorylation in Mice with LPS-Induced ALI

**Figure 8:**
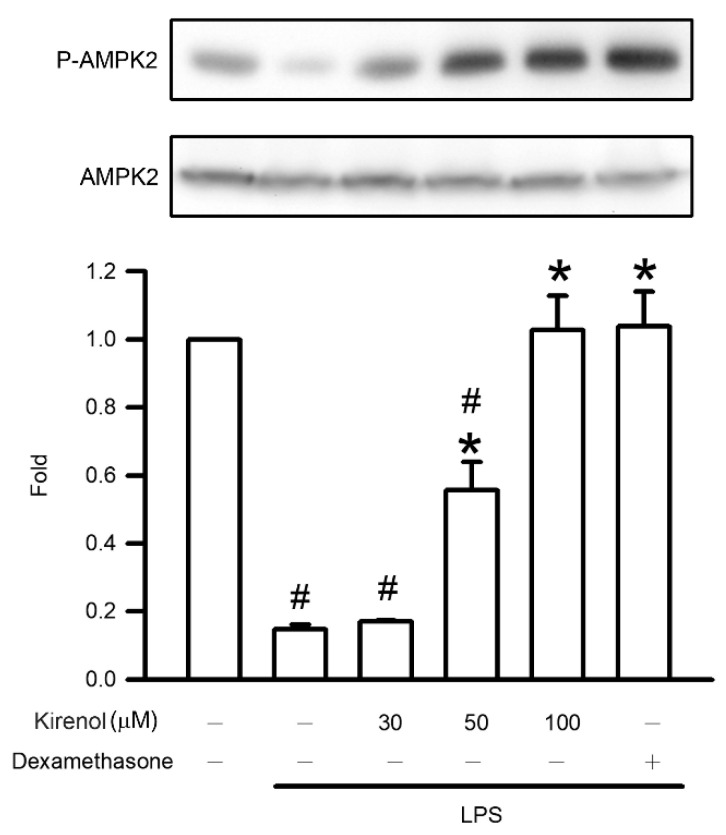
Kirenol attenuated the LPS-reduced the phosphorylation of AMPK2. The levels of AMPK2 phosphorylation in the lung tissues were analyzed by Western blotting analysis. Values are shown the mean ± S.D. of 3–4 mice per group. ^#^ represents the significant difference compared with the Group I (*p* < 0.05); * represents the significant difference compared with Group II (*p* < 0.05).

AMPK2 phosphorylation is an upstream signaling process in the Nrf2/HO-1 pathway. The fold of AMPK2 phosphorylation in lung tissue were 0.15, 0.17, 0.56, 1.03, and 1.04 compared to Group I at the treatment condition of Group II to VI, respectively. AMPK2 phosphorylation was significantly lower in the LPS-treated group than in the control group (*p* < 0.05). Kirenol administration attenuated AMPK2 phosphorylation in a concentration-dependent manner, with significant effects observed starting at 50 mg/kg (*p* < 0.05). Moreover, we also found the mice treatment with dexamethasone attenuated AMPK2 phosphorylation after LPS administration (*p* < 0.05; Figure 8).

### 3.9. Kirenol Suppressed the Leukocyte Infiltration and Alveolar-Capillary Barrier Disruption in Mice with LPS-Induced ALI

**Figure 9:**
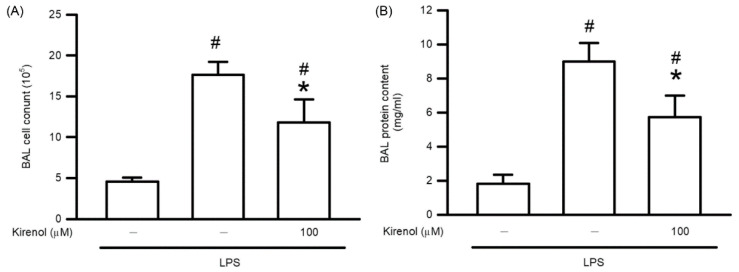
Kirenol suppressed the leukocyte infiltration and alveolar-capillary barrier disruption. (**A**) Leukocyte infiltration was determined by cell counter assay activity in BALF. (**B**) The contents of protein in BALF were measured by Bradford protein analysis. Values are shown the mean ± S.D. of 3–4 mice per group. ^#^ represents the significant difference compared with the Group A (*p* < 0.05); * represents the significant difference compared with Group B (*p* < 0.05).

We further study that the therapeutic effects of kirenol on ALI induced by LPS. Mice received intranasal administration of LPS for 6 h followed by IP injection of kirenol at the concentration of 100 mg/kg for 18 h. As shown in Figure 9, the leukocyte number in BALF were 4.56, 17.63, and 11.79 × 10^5^ at the treatment condition of Group A to C, respectively. The leukocyte number in the BALF of the LPS-treated group was significantly higher than that in the control group (*p* < 0.05). However, post-treatment with kirenol at 100 mg/kg significantly inhibited leukocyte infiltration into the alveolar space (*p* < 0.05). The levels of protein content were 1.82, 9.00, and 5.72 at the treatment condition of Group A to C, respectively. The protein content in the BALF of the LPS-treated group was significantly higher than that in the control group (*p* < 0.05). However, post-treatment with kirenol at 100 mg/kg significantly inhibited plasma protein leakage into the alveolar space (*p* < 0.05).

## 4. Discussion

ALI and its more severe form, the acute respiratory distress syndrome, are the syndromes of pulmonary inflammation that develops in response to influenza viruses, bacterial infection, and even novel infectious diseases such as severe acute respiratory syndrome and coronavirus-19 [20,21]. ALI and acute respiratory distress syndrome involve life-threatening respiratory failure that are associated with high global mortality and morbidity [22,23,24]. Despite its severe impacts, few treatment options are available for patients with ALI [23]. The evidence indicates that kirenol, a major active component isolated from *Herba Siegebeckiae*, has anti-inflammatory benefits for rheumatoid arthritis and diabetic cardiomyopathy [25,26]. The present study investigated the potential protective effect of kirenol on LPS-induced ALI in mice. LPS has been widely used in the establishment of animal models of ALI because it induces symptoms of ALI similar to those in humans, including leukocyte infiltration, alveolar membrane thickening, alveolar space enlargement, and hyaline membrane formation [2,27]. The results demonstrate that kirenol administration mitigated the histopathological impacts of LPS-induced ALI in a concentration-dependent manner, suggesting that kirenol plays the protective reagent against LPS-induced ALI in mice.

The alveolar–capillary membrane serves as the first line of defense against pathogen infection and environmental pollutants in the respiratory tract [28,29,30]. LPS increases its permeability [31]. In the present study, the disruption of this membrane led to protein leakage and leukocyte infiltration into the alveolar space of the mice with LPS-induced ALI [4,7,8]. Kirenol mitigated this disruption in a concentration-dependent manner. In a model of carrageenan-induced rat paw edema, kirenol attenuated the infiltration of inflammatory cells such as neutrophils and eosinophils [32]. To the best of our knowledge, the present study is the first to report that kirenol reduced leukocyte infiltration into the lungs of mice with LPS-induced ALI. In the inflammation of the peripheral tissue, lipid peroxidation was mediated by leukocyte filtration [4,7,8]. A study found that kirenol exerted protective effects on 7,12-dimethylbenz[a]anthracene (DMBA)-induced oral squamous cell carcinogenesis by inhibiting lipid peroxidation [33]. Similarly, in the present study, kirenol suppressed lipid peroxidation in the mice with LPS-induced ALI. The present results suggest that this occurred through the reinforcement of the integrity of the alveolar–capillary membrane and the inhibition of leukocyte infiltration and lipid peroxidation.

Alveolar–capillary membrane disruption and leukocyte activation result from the overexpression of proinflammatory cytokines, including TNFα, IL-1β, and IL-6, in mice with LPS-induced ALI [4,7]. NF-κB activation play the important role in the secretion of these cytokines in the ALI [4,7,8]. In the physiological condition, the IκB binds to the p65, which is the most commonly found component in NF-κB dimmer and retains NF-κB in the cytoplasm. Ubiquitination and degradation of IκB release the NF-κB p65 in turn to phosphorylation and nuclear translocation after LPS stimulation in ALI mice [4,7,8]. Kirenol has been demonstrated to reduce the expression of TNFα, IL-1β, and IL-6 through NF-κB activation in rat models of collagen-induced arthritis, carrageenan-induced paw edema, Freund’s adjuvant-induced paw edema, and formalin-induced paw edema [25,32,34]. Through NF-κB activation, kirenol ameliorates symptoms of photoaging from ultraviolet-B radiation, such as hair loss, wrinkle formation, and skin thickening [35]. In line with the findings from animal studies, kirenol effectively reduced LPS-induced generation of TNFα, IL-1β, and IL-6 in the present study. It also inhibited NF-κB activation. Taken together, the results indicate that kirenol reduced LPS-induced ALI through the inhibition of TNFα, IL-1β, and IL-6 by NF-κB activation.

ROS generation is mediated by leukocytes as a defensive response to pathogen invasion [36]. ROS, in appropriate numbers, are necessary for the elimination of pathogens from the body. However, the lipid peroxidation caused by the overproduction of ROS is associated with various diseases and tissue damage [36]. ROS and relative oxidative stress constitute vital upstream components of NF-κB activation [19,37]. In the present study, kirenol reduced LPS-mediated lipid peroxidation and NF-κB activation in the mice with ALI. These results are consistent with that reported in an animal model of oral squamous cell carcinogenesis that kirenol inhibited lipid peroxidation and NF-κB expression in the oral tissue and plasma of hamsters treated with DMBA [33]. The toxic effects of ROS are reduced by three major AOEs, including SOD, GPx, and catalase [18,19]. SOD converts toxic superoxide anions into hydrogen peroxide, which is then converted by GPx and catalase into the nontoxic products hydrogen oxide and oxygen [19,38]. Kirenol suppressed the lipid peroxidation by reversing the activity of AOEs such as SOD, GPx, and catalase in the skin wounds of diabetic rodents and in the plasma and oral tissue of DMBA-treated hamsters [33,39]. In the present study, kirenol pretreatment significantly downregulated lipid peroxidation and upregulated AOE activity in the lungs of the mice with LPS-induced ALI. In other words, kirenol mitigated pulmonary inflammation by suppressing lipid peroxidation and mitigating the oxidative imbalance in the mice.

Oxidative stress and LPS-induced enzyme HO-1 are responsible for the degradation of heme into carbon monoxide, free iron, and biliverdin [40,41,42]. The conversion of biliverdin into bilirubin is catalyzed by biliverdin reductase. The detoxification of an approximately 10,000-fold excess of lipophilic ROS is mainly achieved through the conversion of bilirubin back into biliverdin [40,41,42]. In the present study, HO-1 expression was induced by kirenol in the mice with LPS-induced ALI in a concentration-dependent manner. Nrf2 is an important upstream transcription factor that modulates the expression of HO-1 and AOEs for ROS detoxification [43]. In the present study, kirenol induced Nrf2 phosphorylation and HO-1 expression in the LPS-challenged mice in a concentration-dependent manner, with significant effects observed starting at 50 mg/kg. In the cellular adaptive response to oxidative stress, AMPK2 leads to the phosphorylation of Nrf2 and then contributes to the expression of HO-1 and AOEs [44]. In the present study, AMPK2 phosphorylation was significantly lower in the lungs of the LPS-challenged mice than in those of the controls. The results suggest that the attenuation of AOE activation downregulation and HO-1 expression through the phosphorylation of Nrf2 and AMPK2 is the critical mechanism by which kirenol alleviates airway inflammation in experimentally induced ALI.

In addition, we further purposed that the therapeutic effects of kirenol on ALI induced by LPS. Kirenol significantly reduced leukocyte infiltration and alveolar-capillary barrier disruption in mice with LPS-induced ALI. Based on these findings, we could purpose that kirenol could be the preventive measure for ALI due to enhanced immunity and lesser toxic effect or fewer side-effects than the modern steroid treatments. Moreover, kirenol has therapeutic effects on ALI. Therefore, kirenol would likely be the novel and effective reagent on the improvement of ALI. Future work should examine the molecular mechanisms of therapeutic exercise in the mice with LPS-induced ALI. Future studies are needed to clarify the treatment strategies in the clinical trials, as well as the molecular mechanisms for the ALI amelioration.

## 5. Conclusions

**Figure 10:**
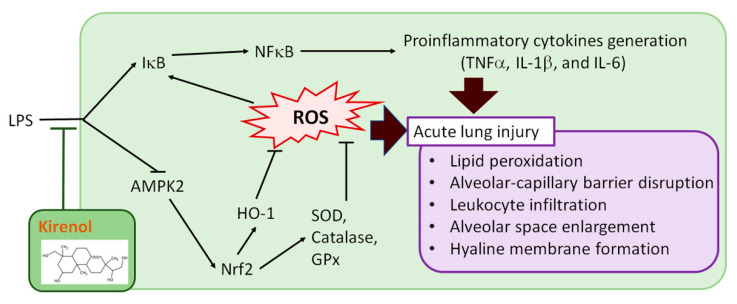
Scheme of the mechanisms in the protective effect of kirenol on LPS-induced ALI.

In conclusion, pretreatment with kirenol reduced the presentation of histopathological changes in mice with LPS-induced ALI, including leukocyte infiltration, alveolar membrane thickening, alveolar space enlargement, hyaline membrane formation, and lipid peroxidation. As shown in Figure 10, the molecular mechanisms underlying the anti-inflammatory effects of kirenol include: (1) the inhibition of proinflammatory cytokines generation, such as TNFα, IL-1β, and IL-6; (2) the reduction of NFκB p65 phosphorylation and IκB degradation; (3) the attenuation of AOEs activities downregulation; (4) the promotion of HO-1 expression and Nrf2 phosphorylation; (5) the attenuation of AMPK2 phosphorylation downregulation. In sum, these present findings indicated that the ameliorative effects of kirenol on the inflammation of LPS-induced ALI mice through inhibiting the proinflammatory NF-κB pathway and promoting the AMPK2-/Nrf2-mediated HO-1/AOE pathway. Thus, we found that the therapeutic effects of kirenol on ALI induced by LPS. Further understanding of the advantageous effects and mechanisms of action of kirenol can aid in the development of treatment strategies for pulmonary diseases characterized by acute inflammation.

## Data Availability

The data presented in this study are available within the article.
